# Influence of magnetoplasmonic γ-Fe_2_O_3_/Au core/shell nanoparticles on low-field nuclear magnetic resonance

**DOI:** 10.1038/srep35477

**Published:** 2016-10-18

**Authors:** Kuen-Lin Chen, Yao-Wei Yeh, Jian-Ming Chen, Yu-Jie Hong, Tsung-Lin Huang, Zu-Yin Deng, Chiu-Hsien Wu, Su-Hsien Liao, Li-Min Wang

**Affiliations:** 1 Department of Physics, National Chung Hsing University, Taichung 402, Taiwan; 2 Institute of Nanoscience, National Chung-Hsing University, Taichung 402, Taiwan; 3 Institute of Electro-Optical Science and Technology, National Taiwan Normal University, Taipei 116, Taiwan; 4 Graduate Institute of Applied Physics and Department of Physics, National Taiwan University, Taipei 106, Taiwan

## Abstract

Magnetoplasmonic nanoparticles, composed of a plasmonic layer and a magnetic core, have been widely shown as promising contrast agents for magnetic resonance imaging (MRI) applications. However, their application in low-field nuclear magnetic resonance (LFNMR) research remains scarce. Here we synthesised γ-Fe_2_O_3_/Au core/shell (γ-Fe_2_O_3_@Au) nanoparticles and subsequently used them in a homemade, high-*T*_c_, superconducting quantum interference device (SQUID) LFNMR system. Remarkably, we found that both the proton spin–lattice relaxation time (T_1_) and proton spin–spin relaxation time (T_2_) were influenced by the presence of γ-Fe_2_O_3_@Au nanoparticles. Unlike the spin–spin relaxation rate (1/T_2_), the spin–lattice relaxation rate (1/T_1_) was found to be further enhanced upon exposing the γ-Fe_2_O_3_@Au nanoparticles to 532 nm light during NMR measurements. We showed that the photothermal effect of the plasmonic gold layer after absorbing light energy was responsible for the observed change in T_1_. This result reveals a promising method to actively control the contrast of T_1_ and T_2_ in low-field (LF) MRI applications.

It is well known that magnetic resonance imaging (MRI) is a powerful, non-invasive technique that has been widely applied to the clinical diagnosis of diseases. However, the quality of magnetic resonance (MR) images is the key for early detection and precise location of infected tissues. MRI is basically based on the measurement of the nuclear magnetic resonance (NMR) signal of water protons. The contrast of MRI is highly dependent on proton density, proton spin–lattice relaxation time (T_1_) and proton spin–spin relaxation time (T_2_). With the aim of improving the image contrast, the so-called contrast agents (CAs) are frequently used in MRI. CAs are generally suspensions of paramagnetic or superparamagnetic nanoparticles, which do not generate NMR signals but influence the relaxation time of nearby protons by altering local magnetic fields. Because of their unique superparamagnetic characteristics, magnetic nanoparticles (MNPs) are commonly used as contrast enhancement agents^1^,^2^,^3^ in MRI applications. Superparamagnetic materials generally exhibit stronger relaxation effects per amount of iron than ferromagnetic particles^4^.

Recently, magnetoplasmonic nanoparticles (i.e. nanoparticles simultaneously showing magnetic and plasmonic characteristics) have become a hot topic of research. Magnetoplasmonic particles (e.g. gold-coated iron oxide nanoparticles) generally consist of a precious metal layer along with a magnetic moiety. The magneto-optical activity of magnetoplasmonic nanoparticles has been shown to be greatly enhanced by the plasmon resonance effect^5^. Because of the good biocompatibility^6^, magnetoplasmonic nanoparticles have also been used in biosensor system^7^, hyperthermia^8^,^9^,^10^ and MRI^10^,^11^,^12^ applications.

Superconducting quantum interference device (SQUID)-based low-field nuclear magnetic resonance (LFNMR) and LFMRI are compelling tools because of their specific advantages^13^. Compared with traditional high-field (HF) systems (magnetic field >1 Tesla), SQUID-based LFNMR devices^13^ are less expensive and have lower operational costs, lower fringe fields, fewer MR artefacts, unique contrast and less energy deposition in tissues. In addition, several groups have demonstrated that LFMRI, either as a standalone technique or integrated with magnetoencephalography (MEG), can successfully image the anatomy of the human brain^14^,^15^,^16^. Espy’s group at Los Alamos National Lab claimed that LFMRI devices are potentially introduced in battlefield scenarios and in developing countries to save lives^17^. Thus, it is expected that LFMRI is progressively introduced as a common clinical technique in the future. However, to satisfy clinical requirements, some characteristics of LFNMR or LFMRI such as signal-to-noise ratio (SNR) and resolution still need to be improved. Therefore, CAs have been used in LFMRI to enhance image contrast^18^,^19^. On the other hand, gold-coated iron oxide nanoparticles had been reported that they are a T_2_ contrast agent with high efficacy in the high field MRI^20^. However, the study on the gold-coated iron oxide nanoparticles as a CA in the low field is still seldom. Here we synthesised γ-Fe_2_O_3_/Au core/shell (γ-Fe_2_O_3_@Au) nanoparticles and subsequently used them in a homemade, high-*T*_c_, SQUID-based LFNMR system with the aim of studying the effect of magnetoplasmonic nanoparticles over the proton NMR relaxation time, while maintaining in LF regimes. The details of our homemade, high-*T*_c_, SQUID-based LFNMR system can be found elsewhere^21^. We found that both T_1_ and T_2_ were affected by the presence of γ-Fe_2_O_3_@Au nanoparticles. Furthermore, the spin–lattice relaxation rate (1/T_1_) and spin–spin relaxation rate (1/T_2_) varied with the concentration of nanoparticles. Remarkably, unlike 1/T_2_, 1/T_1_ was further enhanced under exposure of γ-Fe_2_O_3_@Au nanoparticles to 532 nm light, thereby revealing a promising method to actively control the contrast of T_1_ and T_2_ images during NMR measurements.

## Results and Discussions

**Figure 1:**
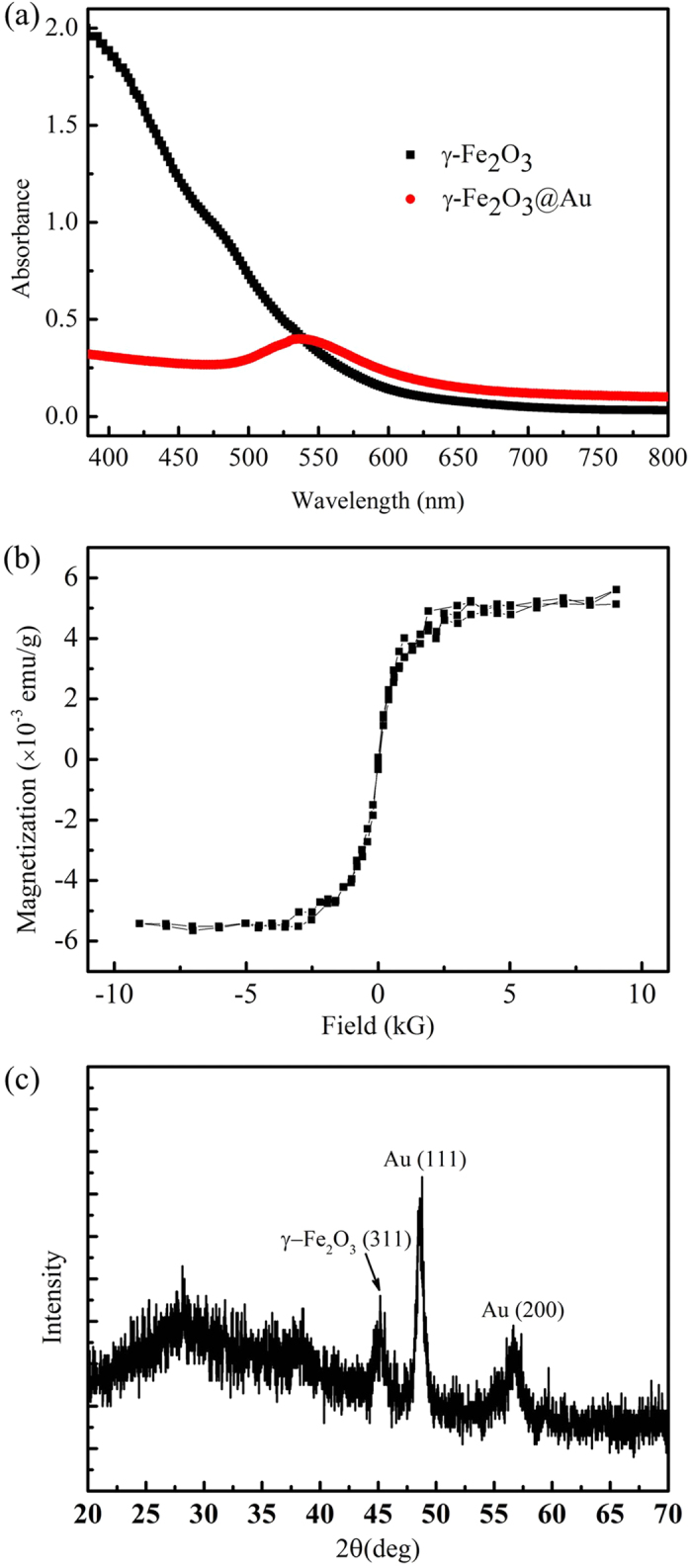
(**a**) UV–Vis absorption spectrum of the *γ*-Fe_2_O_3_ and γ-Fe_2_O_3_@Au nanoparticles. The plasmonic absorption peak of the γ-Fe_2_O_3_@Au nanoparticles is approximately 536 nm. (**b**) Hysteresis curve of the γ-Fe_2_O_3_@Au nanoparticles measured by the SQUID MPMS system at 300 K. (**c**) The powder XRD pattern of the synthesised γ-Fe_2_O_3_@Au nanoparticles.

**Figure 2:**
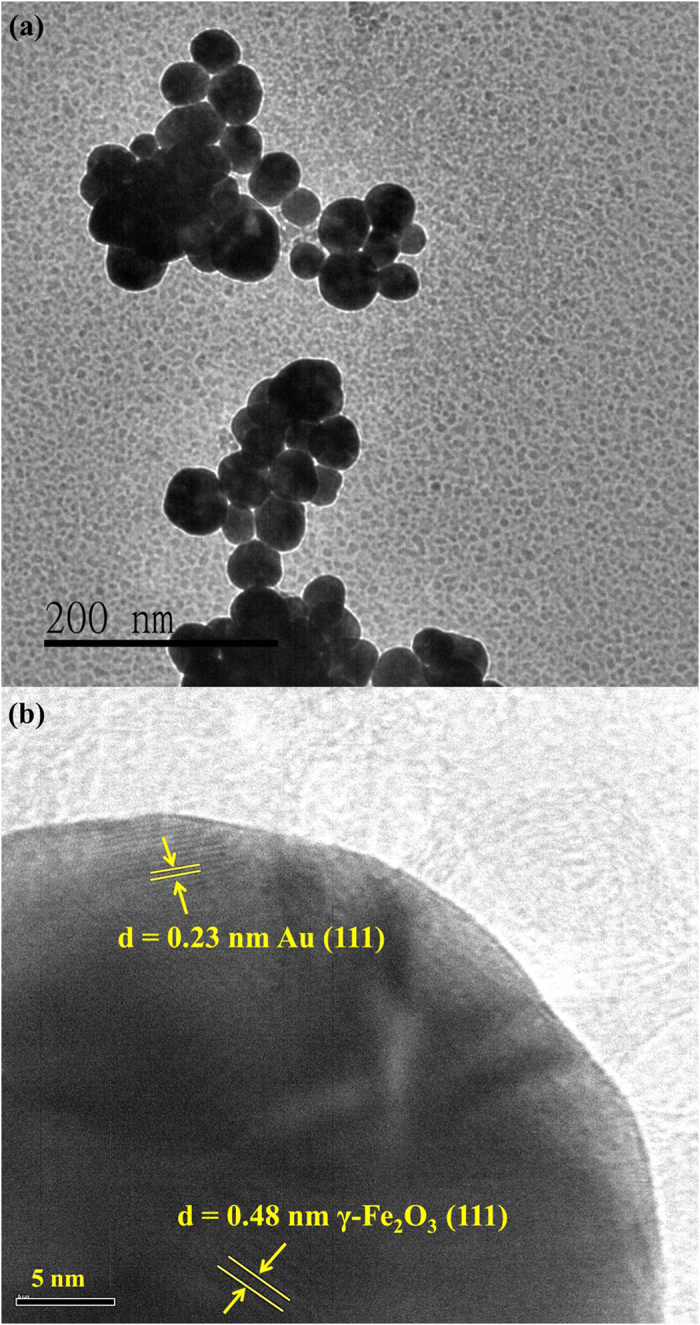
HRTEM images of the γ-Fe_2_O_3_@Au nanoparticles. (**a**) A low-magnification TEM image of the γ-Fe_2_O_3_@Au nanoparticles. (**b**) Locally magnified image of a γ-Fe_2_O_3_@Au nanoparticle. The Au shell and *γ*-Fe_2_O_3_ core crystal structures are observed.

The average hydrodynamic size (i.e. diameter) of the synthesised γ-Fe_2_O_3_@Au nanoparticles, determined by dynamic light scattering (DLS) (Nanotrac-150, Microtrac), was 28.38 ± 6.26 nm. To increase the reliability of particle size, the average hydrodynamic size of the γ-Fe_2_O_3_@Au nanoparticles was calculated from the DLS measurement data of ten samples. Figure 1a shows the UV–Vis absorption spectrum of both *γ*-Fe_2_O_3_ and γ-Fe_2_O_3_@Au nanoparticles. A characteristic plasmon peak at 536 nm, arising from the contribution of localised surface plasmon resonance (LSPR) of the Au shell, can be clearly observed in the case of hybrid nanoparticles. Figure 1b shows the hysteresis curve of γ-Fe_2_O_3_@Au nanoparticles measured by a SQUID magnetic property measurement system (MPMS, Quantum Design, Inc) at 300 K. The hysteresis curve of γ-Fe_2_O_3_@Au nanoparticles is characteristic of superparamagnetic materials with a saturation field of 2500 Gauss. Figure 1c is the powder X-ray diffraction (XRD) pattern (LabX XRD-6000, Shimadzu; iron−K radiation 0.1936 nm) of the synthesised γ-Fe_2_O_3_@Au nanoparticles. The XRD pattern shows peaks corresponding to known lattice planes of Au and γ-Fe_2_O_3_ and verifies that the synthesised nanoparticles contain Au and γ-Fe_2_O_3_ phases. Furthermore, high-resolution transmission electron microscopy (HRTEM) (JEM-2010, JEOL Co. Ltd) was used to confirm the structure of γ-Fe_2_O_3_@Au nanoparticles. Figure 2a is a low-magnification TEM image of the γ-Fe_2_O_3_@Au nanoparticles and it shows that γ-Fe_2_O_3_@Au nanoparticles are nearly spherical with a diameter of approximately 30~50 nm. A magnified TEM image (Fig. 2b) reveals two different crystal structures, that is, *γ*-Fe_2_O_3_ (111) in the core and Au (111) near the surface, thereby confirming the core–shell nature of the synthesised γ-Fe_2_O_3_@Au nanoparticles.

Our homemade, high-*T*_c_, SQUID-based LFNMR system was used to investigate the effect of γ-Fe_2_O_3_@Au nanoparticles over T_1_ and T_2_ proton relaxation times. The LFNMR measurements were performed by utilizing a pulsed pre-polarisation magnetic field of 70 mT and a measuring field of 100 μT. However, in this experiment, one should note that the effective background fields of T_1_ and T_2_ relaxations are different because of the different NMR measurement sequences. The details on the measurement sequences of T_1_ relaxation^22^ and T_2_ relaxation^23^ can be found elsewhere^22^,^23^. The volume of experimental sample was 1 mL. With the aim of quantifying the contribution of LSPR effect of γ-Fe_2_O_3_@Au nanoparticles to the global proton NMR signal, the sample was irradiated with 532 nm green laser light (100 μW power) via an optical fibre. The whole measurement process was performed at a stable temperature of 298 K, as confirmed by measuring the temperature before and after the irradiation.

**Figure 3:**
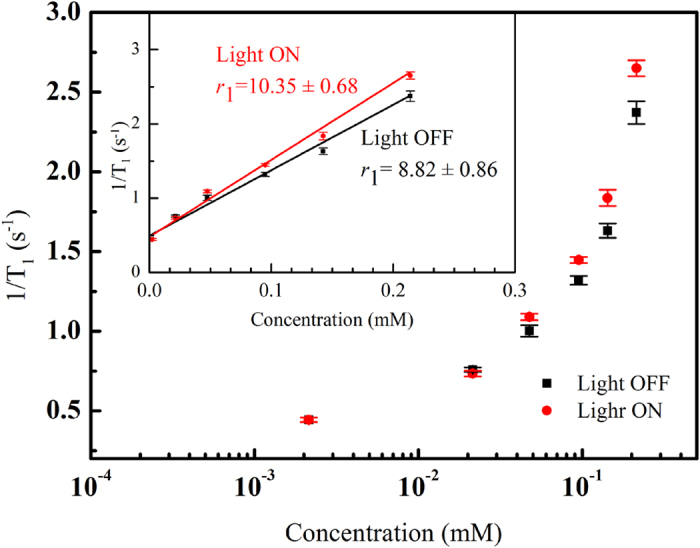
Spin–lattice relaxation rate (1/T_1_) as a function of iron concentration in a 1 mL aqueous solution of *γ*-Fe_2_O_3_@Au nanoparticles in the presence and absence of light irradiation. The insert shows the longitudinal relaxivity of the *γ*-Fe_2_O_3_@Au nanoparticles. The values are presented as the mean ± SEM after 10 measurements.

**Figure 4:**
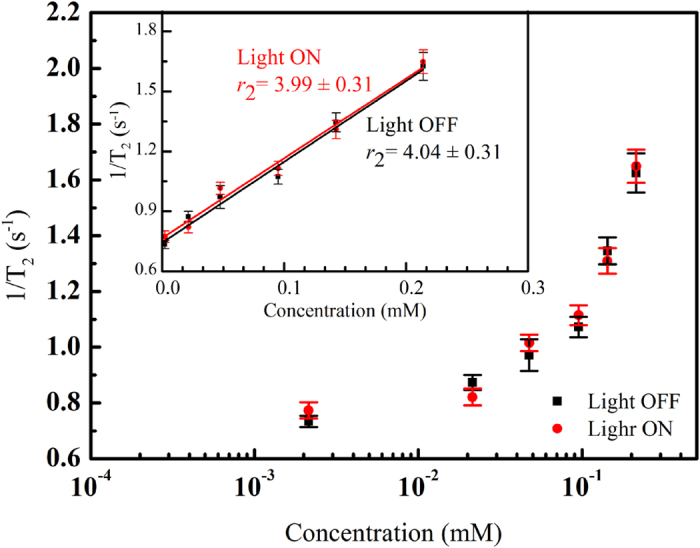
Spin–spin relaxation rate (1/T_2_) as a function of iron concentration of a 1 mL *γ*-Fe_2_O_3_@Au nanoparticles aqueous solution in the presence and absence of light irradiation. The insert shows the transverse relaxivity of the *γ*-Fe_2_O_3_@Au nanoparticles. The values are also presented as the mean ± SEM after 10 measurements.

Figures 3 and 4 show 1/T_1_ and 1/T_2_, respectively, as a function of iron concentration in an aqueous suspension of *γ*-Fe_2_O_3_@Au nanoparticles (1 mL) in the presence and absence of light irradiation. The number of replicates for all the measurements was 10, and detailed data (expressed as mean ± standard error of the mean, SEM) are shown in Table 1 and Table 2. As can be clearly seen, 1/T_1_ changed upon light irradiation, with this variation being statistically significant (P < 0.05, t-test calculation) at iron concentrations higher than 0.048 mM. Remarkably, the longitudinal relaxivity (*r*_1_) of *γ*-Fe_2_O_3_@Au nanoparticles was enhanced as a result of light exposure (10.3 vs 8.81 mM^−1^ s^−1^). Conversely, 1/T_2_ was found to be unaffected by light irradiation regardless of the ion concentration because the variances of this parameter were not statistically significant in all cases. In addition, the transverse relaxivity (*r*_2_) was found to be nearly unchanged (approximately 4.0 mM^−1^ s^−1^) with irradiation conditions.

We speculate that the photothermal effect of the plasmonic gold layer under light energy absorption could mainly account for the observed change in T_1_. The plasmonic photothermal therapy based on gold nanoparticles has been widely studied as a non-invasive cancer treatment^24^. The special plasmonic characteristics of gold nanoparticles allow photon light energy to be effectively absorbed and subsequently converted to heat. It is well known that temperature affects the proton spin relaxation. We previously found 1/T_1_ and 1/T_2_ proton relaxation rates to decrease with temperature in a magnetic fluid as a result of the enhanced Brownian motion of MNPs^25^. Therefore, the enhancement of 1/T_1_ relaxation rate cannot be attributed to the temperature increasing by laser irradiation. Moreover, here we ensured constant sample temperature using very-low-power laser irradiation (100 μW) during the experiment, although it seems that this energy is still high enough to influence the motion of magnetic *γ*-Fe_2_O_3_@Au nanoparticles. As indicated above, we did not observe variation of T_2_ under light irradiation. It means that the light irradiation does not induce the temperature fluctuation in the sample.

On the other hand, T_1_ is dominated by the interaction between the proton spin and the surrounding lattice. The relaxation of water protons mainly depends on the dipolar coupling between the magnetic moments of water protons and magnetic particles^26^. Zhou *et al*.^27^ showed that the main contribution of the T_1_ contrast of magnetic nanoplates is the chemical exchange on the iron-rich Fe_3_O_4_(111) surfaces, which happens in the innershpere regime. They also showed that surface coating layer of nanoplates will hinder the chemical exchanges between the surrounding protons and surface iron metals. Referring to Zhou’s work, we think that the T_1_ shortening effect in our system is due to the outersphere translational diffusion of protons because the gold layer of the *γ*-Fe_2_O_3_@Au nanoparticles would hinder the chemical exchange happening in the innersphere regime. We inferred that the motion of the *γ*-Fe_2_O_3_@Au nanoparticles became more active after light irradiation. Thus, a greater energetic motion of the *γ*-Fe_2_O_3_@Au nanoparticles would increase the collision rate with surrounding water protons. Energetic collision would shorten the distance between the magnetic moments of water protons and the *γ*-Fe_2_O_3_@Au nanoparticles and enhance their correlation. Increasing collision rate would also reduce the effective diffusion length of outersphere water protons and hence increase the diffusion correlation time. The enhanced correlation and the increased diffusion correlation time both facilitate the proton spins to effectively release their rf pulse magnetic energy back to the surrounding and shorten T_1_ after light irradiation.

**Figure 5:**
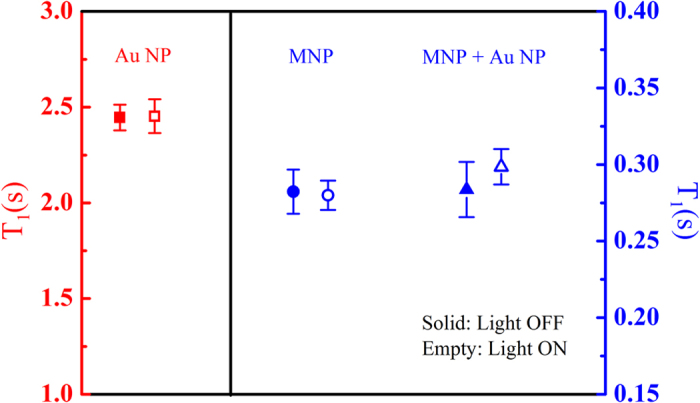
T_1_ values of the aqueous solutions containing Au nanoparticles (NP), Fe_3_O_4_ magnetic nanoparticles (MNP) and a mixture of Au and Fe_3_O_4_ MNP in the presence and absence of light irradiation. The empty and solid symbols represent the data measured in the presence and absence of light irradiation, respectively. The data of Au NPs refer to the left axis, whereas the data of MNPs and the mixture of Au NPs and MNPs are represented in the right axis. These values are also presented as the mean ± SEM after 10 measurements.

To confirm our assumption, we measured T_1_ of aqueous solutions containing Au nanoparticles, Fe_3_O_4_ magnetic nanoparticles and a mixture of Au and Fe_3_O_4_ nanoparticles (Table 3) under light irradiation at a constant temperature (i.e. temperature was unchanged during irradiation). The iron and gold concentrations in the solutions are 0.106 mM and 0.13 mM, respectively. The gold concentration is twice the gold concentration of the *γ*-Fe_2_O_3_@Au nanoparticles with the same iron concentration. As shown in Fig. 5, T_1_ values remained constant after light irradiation for the three samples. In the case of light-absorbing Au nanoparticles, the low light intensity used prevented this sample from heating, thereby leaving T_1_ unaltered. In the case of the sample Fe_3_O_4_ nanoparticles, T_1_ is likely remained constant after irradiation because MNPs do not intrinsically absorb light energy. In the case of the sample containing a mixture of nanoparticles, both Au and magnetic entities were independently suspended in the solution, leading to ineffective motion excitement of MNPs by light-absorbing Au nanoparticles. Therefore, the correlation between water protons and MNPs cannot be effectively enhanced by light irradiation, and the T_1_ relaxation time thus remained unchanged. The results showed that even the solution with higher concentration of Au nanoparticle, it still cannot induce the enhancement of 1/T_1_ relaxation rate under light irradiation. Only the core–shell *γ*-Fe_2_O_3_@Au nanoparticles can effectively enhance the 1/T_1_ relaxation rate under light irradiation. These results strongly support our assumptions and prove that the unique core–shell structure of the *γ*-Fe_2_O_3_@Au nanoparticles allows control of T_1_ by light irradiation within the LFNMR system.

In summary, we synthesised core–shell *γ*-Fe_2_O_3_@Au nanoparticles to be used in LFNMR systems. We found that the presence of *γ*-Fe_2_O_3_@Au nanoparticles in the aqueous solution can effectively modify T_1_ and T_2_. In particular, T_1_ can be further altered by the irradiation with 532 nm light, and the alteration was produced by energetic motion of *γ*-Fe_2_O_3_@Au nanoparticles induced by a photothermal effect. Because a mixture of individual Au and MNPs was not effective in changing T_1_, we concluded that the unique magnetoplasmonic characteristics of *γ*-Fe_2_O_3_@Au nanoparticles are responsible for this behaviour. This study thus offers a promising way to modulate the contrast of LFMRI devices by light irradiation with *γ*-Fe_2_O_3_@Au nanoparticles as CAs.

## Methods

### Synthesis of the core–shell *γ*-Fe_2_O_3_@Au nanoparticles

FeCl_3_.6H_2_O and FeCl_2_.4H_2_O powders were initially dissolved in water (2:1). NaOH solution was then added into the ferric solution under vigorous stirring. Ferric ions were reduced to Fe_3_O_4_ magnetic particles, resulting in the formation of a black precipitate, which was washed twice with ultrapure water. A 0.1 M HNO_3_ solution was subsequently added to wash the Fe_3_O_4_ nanoparticles, and the resulting solution was centrifuged at 6,000 rpm for 20 min.

The precipitate was collected and re-dissolved in 0.01 M HNO_3_ at 90 °C–100 °C (water bath) under continuous stirring for 30 min and subsequently oxidised to γ-Fe_2_O_3_ to form a reddish brown colloidal solution. γ-Fe_2_O_3_ particles were collected by centrifugation (6,000 rpm, 20 min) and washed twice with ultrapure water.

The nanoparticle suspension was formed using a 36 mM tetramethylammonium hydroxide [TMAOH, N(CH_3_)_4_OH] solution at pH = 12. TMAOH dissociation results in the release of N(CH_3_)^+^ and OH^−^ ions; the latter allow suspension by linking to the surface of γ-Fe_2_O_3_ nanoparticles.

The suspending solution of γ-Fe_2_O_3_ nanoparticles was diluted to 0.1 mM, and an equal volume of a 0.1 M sodium citrate (C_6_H_5_O_7_Na_3_.2H_2_O) aqueous solution was added. The resulting solution was stirred for at least 10 min, during which the citrate ion (C_6_H_5_O_7_^3−^) replaced OH^−^ on the surface of γ-Fe_2_O_3_ nanoparticles. The γ-Fe_2_O_3_ nanoparticle colloidal solution was subsequently diluted 20-fold to reach a 2.5 mM citrate salt concentration, and an equal volume of tetrachloroauric acid (1% HAuCl_4_) solution was prepared for iterative seeding process. The seeding process consisted of a slow addition of NH_2_OH·HCl and HAuCl_4_ in excess to the γ-Fe_2_O_3_ nanoparticle colloidal solution. This seeding process was repeated (seeding time higher than 10 s) four times, with at least a 10-min interval between iterations. This process allows Au^3+^ ions to reduce and subsequently link on the surface of the nanoparticles. The colour of the solution shifted from purple to deep pink as the Au layer was formed on the surface of the magnetic particles.

## Additional Information

**How to cite this article**: Chen, K.-L. *et al*. Influence of magnetoplasmonic γ-Fe_2_O_3_/Au core/shell nanoparticles on low-field nuclear magnetic resonance. *Sci. Rep.* **6**, 35477; doi: 10.1038/srep35477 (2016).

## Figures and Tables

**Table 1:** Spin–lattice relaxation rate (1/T_1_) of 1 mL *γ*-Fe_2_O_3_@Au aqueous solution with and without light irradiation.

1/T_1_ (s^−1^)						
Iron concentration (mM)	0.21	0.14	0.095	0.048	0.021	0.0021
Light OFF	2.372 ± 0.071	1.631 ± 0.045	1.320 ± 0.027	1.003 ± 0.036	0.758 ± 0.014	0.444 ± 0.013
Light On	2.651 ± 0.050	1.835 ± 0.051	1.447 ± 0.019	1.090 ± 0.020	0.734 ± 0.017	0.444 ± 0.014
P-value	0.0016	0.0032	0.0005	0.0379	0.1693	0.4950

The data are presented as the mean ± SEM from 10 measurements. The P-value is based on single-trail paired t-test calculation.

**Table 2:** Spin–spin relaxation rate (1/T_2_) of 1 mL *γ*-Fe_2_O_3_@Au aqueous solution with and without light irradiation.

1/T_2_ (s^−1^)						
Iron concentration (mM)	0.21	0.14	0.095	0.048	0.021	0.0021
Light OFF	1.625 ± 0.086	1.345 ± 0.059	1.072 ± 0.045	0.971 ± 0.069	0.873 ± 0.032	0.734 ± 0.025
Light On	1.648 ± 0.073	1.309 ± 0.056	1.115 ± 0.044	0.998 ± 0.042	0.822 ± 0.036	0.773 ± 0.036
P-value	0.4197	0.3357	0.2591	0.3765	0.1521	0.2105

The data are presented as the mean ± SEM from 10 measurements. The P-value is based on single-trail paired t-test calculation.

**Table 3:** T_1_ values of Au nanoparticles, Fe_3_O_4_ magnetic nanoparticles and mixture of Au and Fe_3_O_4_ magnetic nanoparticles (aqueous solutions) with and without light irradiation.

T_1_ (s)	Au nanoparticle		Magnetic nanoparticle		Magnetic nanoparticle + Au nanoparticle	
Light	Off	On	Off	On	Off	On
Average	2.447	2.453	0.282	0.280	0.284	0.299
SEM	0.067	0.088	0.014	0.010	0.018	0.011
P-value	0.469		0.247		0.448	

The data are presented as the mean ± SEM from 10 measurements. The P-value is based on single-trail paired t-test calculation.
